# Poly[tetra­kis­(μ-benzene-1,2-dicarboxyl­ato)di-μ-formato-penta­strontium(II)]

**DOI:** 10.1107/S1600536811044977

**Published:** 2011-11-02

**Authors:** Pei-Chi Cheng, Jun-Xiang Zhan, Cheng-You Wu, Chia-Her Lin

**Affiliations:** a Department of Chemistry, R&D Center for Membrane Technology, Center for Nanotechnology, Chung-Yuan Christian University, Chung-Li 320, Taiwan; b Department of Chemistry, Chung-Yuan Christian University, Chung-Li 320, Taiwan

## Abstract

The asymmetric unit of the title complex, [Sr_5_(C_8_H_4_O_4_)_4_(HCO_2_)_2_]_*n*_, contains three independent Sr^II^ ions, one of which is located on an inversion center. In the crystal, the Sr^II^ ions (coordination numbers 8, 9 and 12) are connected by two crystallographically distinct benzene-1,2-dicarboxyl­ate ligands and one formate ligand, forming a two-dimensional polymer parallel to (001).

## Related literature

For general background to metal coordination polymers, see: Kitagawa *et al.* (2004 ▶). For related structures, see: Stein & Ruschewitz (2005 ▶); Zhang *et al.* (2009 ▶); Wang *et al.* (2010 ▶). 
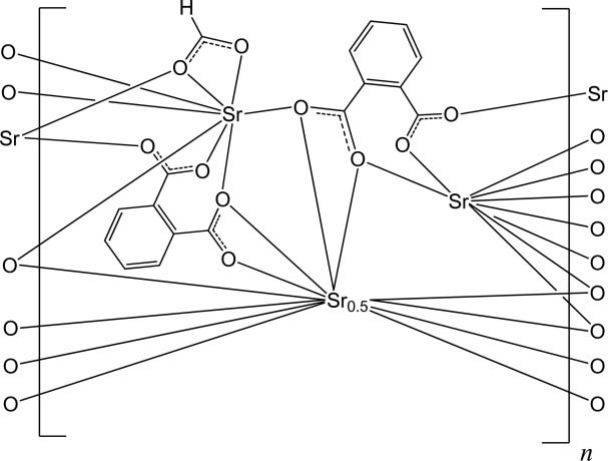


## Experimental

### 

#### Crystal data

[Sr_5_(C_8_H_4_O_4_)_4_(HCO_2_)_2_]
*M* *_r_* = 1184.58
Triclinic, 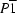

*a* = 7.0292 (3) Å
*b* = 10.2892 (4) Å
*c* = 12.5439 (5) Å
α = 91.361 (2)°
β = 90.407 (2)°
γ = 104.998 (2)°
*V* = 876.00 (6) Å^3^
*Z* = 1
Mo *K*α radiation
μ = 7.65 mm^−1^
*T* = 295 K
0.20 × 0.18 × 0.15 mm

#### Data collection

Bruker APEXII CCD diffractometer
Absorption correction: multi-scan (*SADABS*; Bruker, 2010 ▶) *T*_min_ = 0.310, *T*_max_ = 0.393
15465 measured reflections
4295 independent reflections
3585 reflections with *I* > 2σ(*I*)
*R*_int_ = 0.033

#### Refinement

*R*[*F* ^2^ > 2σ(*F* ^2^)] = 0.028
*wR*(*F* ^2^) = 0.060
*S* = 1.04
4295 reflections
268 parameters
H-atom parameters constrained
Δρ_max_ = 0.97 e Å^−3^
Δρ_min_ = −0.39 e Å^−3^

### 

Data collection: *APEX2* (Bruker, 2010 ▶); cell refinement: *SAINT* (Bruker, 2010 ▶); data reduction: *SAINT*; program(s) used to solve structure: *SHELXS97* (Sheldrick, 2008 ▶); program(s) used to refine structure: *SHELXL97* (Sheldrick, 2008 ▶); molecular graphics: *DIAMOND* (Brandenburg, 2010 ▶); software used to prepare material for publication: *SHELXTL* (Sheldrick, 2008 ▶).

## Supplementary Material

Crystal structure: contains datablock(s) I, global. DOI: 10.1107/S1600536811044977/lh5360sup1.cif

Structure factors: contains datablock(s) I. DOI: 10.1107/S1600536811044977/lh5360Isup2.hkl

Additional supplementary materials:  crystallographic information; 3D view; checkCIF report

## Figures and Tables

**Fig. 1.**
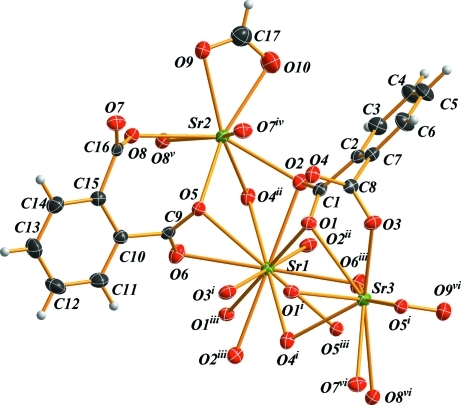
Part of the title structure, showing 50% probability displacement ellipsoids. [symmetry codes: (i) 1 - x, -y, -z; (ii) 1 + x, y, z; (iii) 2 - x, -y, -z; (iv) 1 - x, -1 - y, -z; (v) 2 - x, -1 - y, -z; (vi) x, 1 + y, z].

**Fig. 2.**
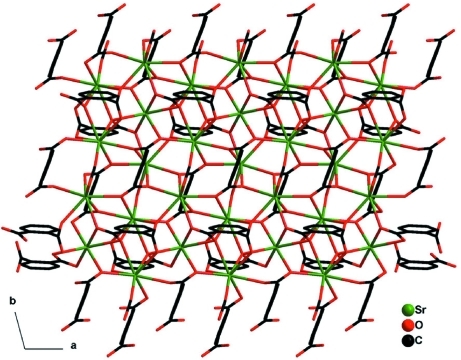
The layer structure of the title compound viewed along the c axis.

## supplementary crystallographic information

### Comment

The increasingly rapid development of metal coordination polymers over the past two decades has attracted considerable attention due to their structural diversity and important applications (Kitagawa *et al.*, 2004). benzene-1,2-dicarboxylate acid (H_2_BDC) has been successively applied to construct to strontium (Stein & Ruschewitz, 2005), lead (Zhang *et al.*, 2009), and tin complexes (Wang *et al.*, 2010). Here we report the crystal structure of the title complex.

The title compound contains three crystallographically independent Sr^II^ ions, with coordination numbers 12 (Sr1, located on an inversion center), 8 (Sr2) and 9 (Sr3). The Sr—O distances range from 2.467 (2) to 2.9332 (19) Å. The coordination geometry of the Sr(II) ions is shown in Fig. 1. In the crystal, the Sr^II^ ions are connected by two crystallographically distinct benzene-1,2-dicarboxylate ligands and one formate ligand, to form a two-dimensional polymer parallel to (001) [Fig. 2].

### Experimental

Solvothermal reactions were carried out at 423 K for 2 d in a Teflon-lined acid digestion bomb with an internal volume of 23 ml followed by slow cooling at 6 K/h to room temperature. A single-phase product consisting of transparent colorless crystals of was obtained from a mixture of Sr(NO_3_)_2_ (0.0847 g,0.4 mmol), H_2_ortho-BDC (0.0332 g, 0.2 mmol), DMF (5.0 ml) and H_2_O (1.0 ml).

### Refinement

H atoms were placed in ideal geometries, with C—H = 0.93 Å and *U*_iso_(H)= 1.2*U*_eq_(C).

### Crystal data

**Table:**

[Sr_5_(C_8_H_4_O_4_)_4_(HCO_2_)_2_]	*Z* = 1
*M**_r_* = 1184.58	*F*(000) = 572
Triclinic, *P*1	*D*_x_ = 2.245 Mg m^−^^3^
Hall symbol: -P 1	Mo *K*α radiation, λ = 0.71073 Å
*a* = 7.0292 (3) Å	Cell parameters from 8942 reflections
*b* = 10.2892 (4) Å	θ = 2.6–28.3°
*c* = 12.5439 (5) Å	µ = 7.65 mm^−^^1^
α = 91.361 (2)°	*T* = 295 K
β = 90.407 (2)°	Lamellar, colorless
γ = 104.998 (2)°	0.20 × 0.18 × 0.15 mm
*V* = 876.00 (6) Å^3^	

### Data collection

**Table:**

Bruker APEXII CCD diffractometer	4295 independent reflections
Radiation source: fine-focus sealed tube	3585 reflections with *I* > 2σ(*I*)
graphite	*R*_int_ = 0.033
Detector resolution: 8.3333 pixels mm^-1^	θ_max_ = 28.3°, θ_min_ = 1.6°
φ and ω scans	*h* = −9→9
Absorption correction: multi-scan (*SADABS*; Bruker, 2010)	*k* = −13→13
*T*_min_ = 0.310, *T*_max_ = 0.393	*l* = −16→16
15465 measured reflections	

### Refinement

**Table:**

Refinement on *F*^2^	Primary atom site location: structure-invariant direct methods
Least-squares matrix: full	Secondary atom site location: difference Fourier map
*R*[*F*^2^ > 2σ(*F*^2^)] = 0.028	Hydrogen site location: inferred from neighbouring sites
*wR*(*F*^2^) = 0.060	H-atom parameters constrained
*S* = 1.04	*w* = 1/[σ^2^(*F*_o_^2^) + (0.0254*P*)^2^ + 0.4547*P*] where *P* = (*F*_o_^2^ + 2*F*_c_^2^)/3
4295 reflections	(Δ/σ)_max_ = 0.001
268 parameters	Δρ_max_ = 0.97 e Å^−^^3^
0 restraints	Δρ_min_ = −0.39 e Å^−^^3^

### Special details

**Table:**

Geometry. All e.s.d.'s (except the e.s.d. in the dihedral angle between two l.s. planes) are estimated using the full covariance matrix. The cell e.s.d.'s are taken into account individually in the estimation of e.s.d.'s in distances, angles and torsion angles; correlations between e.s.d.'s in cell parameters are only used when they are defined by crystal symmetry. An approximate (isotropic) treatment of cell e.s.d.'s is used for estimating e.s.d.'s involving l.s. planes.
Refinement. Refinement of *F*^2^ against ALL reflections. The weighted *R*-factor *wR* and goodness of fit *S* are based on *F*^2^, conventional *R*-factors *R* are based on *F*, with *F* set to zero for negative *F*^2^. The threshold expression of *F*^2^ > σ(*F*^2^) is used only for calculating *R*-factors(gt) *etc*. and is not relevant to the choice of reflections for refinement. *R*-factors based on *F*^2^ are statistically about twice as large as those based on *F*, and *R*- factors based on ALL data will be even larger.

### Fractional atomic coordinates and isotropic or equivalent isotropic displacement parameters (Å^2^)

**Table:**

	*x*	*y*	*z*	*U*_iso_*/*U*_eq_	
Sr1	1.0000	0.0000	0.0000	0.01600 (9)	
Sr2	0.86724 (3)	−0.37889 (2)	−0.11377 (2)	0.01683 (7)	
Sr3	0.57129 (3)	0.21010 (2)	−0.05482 (2)	0.01681 (7)	
O1	0.6246 (3)	−0.05156 (18)	−0.08904 (15)	0.0184 (4)	
O2	0.8372 (3)	−0.14323 (19)	−0.17205 (16)	0.0219 (4)	
O3	0.2714 (3)	0.03708 (19)	−0.14874 (16)	0.0227 (4)	
O4	0.1994 (3)	−0.17952 (19)	−0.11074 (16)	0.0209 (4)	
O5	0.7838 (3)	−0.26762 (19)	0.05581 (15)	0.0215 (4)	
O6	1.0850 (3)	−0.18764 (19)	0.12185 (17)	0.0254 (5)	
O7	0.5072 (3)	−0.61995 (19)	0.10329 (16)	0.0233 (4)	
O8	0.8151 (3)	−0.54513 (18)	0.05059 (15)	0.0190 (4)	
O9	0.6767 (3)	−0.6166 (2)	−0.19012 (17)	0.0274 (5)	
O10	0.8059 (4)	−0.4615 (3)	−0.30783 (19)	0.0432 (6)	
C1	0.6836 (4)	−0.1025 (3)	−0.1717 (2)	0.0168 (6)	
C2	0.5647 (4)	−0.1157 (3)	−0.2734 (2)	0.0178 (6)	
C3	0.6460 (4)	−0.1431 (3)	−0.3689 (2)	0.0268 (7)	
H3A	0.7758	−0.1489	−0.3701	0.032*	
C4	0.5356 (5)	−0.1618 (3)	−0.4624 (3)	0.0316 (7)	
H4A	0.5917	−0.1788	−0.5265	0.038*	
C5	0.3424 (5)	−0.1553 (3)	−0.4603 (3)	0.0327 (8)	
H5A	0.2678	−0.1684	−0.5231	0.039*	
C6	0.2587 (4)	−0.1293 (3)	−0.3652 (3)	0.0292 (7)	
H6A	0.1279	−0.1256	−0.3643	0.035*	
C7	0.3690 (4)	−0.1087 (3)	−0.2717 (2)	0.0184 (6)	
C8	0.2747 (4)	−0.0818 (3)	−0.1693 (2)	0.0172 (6)	
C9	0.9131 (4)	−0.2604 (3)	0.1291 (2)	0.0166 (6)	
C10	0.8555 (4)	−0.3394 (3)	0.2271 (2)	0.0183 (6)	
C11	0.9164 (4)	−0.2784 (3)	0.3253 (3)	0.0280 (7)	
H11A	0.9963	−0.1909	0.3287	0.034*	
C12	0.8602 (5)	−0.3458 (3)	0.4187 (3)	0.0336 (8)	
H12A	0.9028	−0.3042	0.4843	0.040*	
C13	0.7407 (5)	−0.4750 (3)	0.4136 (3)	0.0343 (8)	
H13A	0.7023	−0.5208	0.4760	0.041*	
C14	0.6777 (4)	−0.5367 (3)	0.3161 (3)	0.0275 (7)	
H14A	0.5956	−0.6236	0.3136	0.033*	
C15	0.7349 (4)	−0.4711 (3)	0.2222 (2)	0.0180 (6)	
C16	0.6787 (4)	−0.5489 (3)	0.1181 (2)	0.0159 (6)	
C17	0.7073 (5)	−0.5772 (4)	−0.2845 (3)	0.0377 (8)	
H17A	0.6537	−0.6374	−0.3400	0.045*	

### Atomic displacement parameters (Å^2^)

**Table:**

	*U*^11^	*U*^22^	*U*^33^	*U*^12^	*U*^13^	*U*^23^
Sr1	0.01285 (16)	0.01455 (18)	0.0203 (2)	0.00311 (13)	−0.00091 (13)	0.00027 (15)
Sr2	0.01502 (12)	0.01474 (13)	0.02072 (15)	0.00390 (10)	0.00023 (10)	0.00003 (10)
Sr3	0.01324 (12)	0.01526 (13)	0.02143 (15)	0.00273 (9)	0.00058 (10)	0.00130 (10)
O1	0.0151 (9)	0.0180 (10)	0.0201 (11)	0.0006 (8)	−0.0004 (8)	−0.0016 (8)
O2	0.0151 (9)	0.0235 (11)	0.0280 (12)	0.0065 (8)	−0.0020 (8)	0.0008 (9)
O3	0.0229 (10)	0.0179 (10)	0.0287 (12)	0.0080 (8)	−0.0001 (9)	−0.0042 (9)
O4	0.0140 (9)	0.0210 (10)	0.0263 (12)	0.0015 (8)	0.0003 (8)	0.0058 (9)
O5	0.0269 (10)	0.0175 (10)	0.0216 (11)	0.0086 (8)	−0.0045 (8)	−0.0001 (8)
O6	0.0189 (10)	0.0213 (11)	0.0352 (13)	0.0027 (8)	0.0041 (9)	0.0083 (9)
O7	0.0153 (9)	0.0236 (11)	0.0287 (12)	0.0013 (8)	−0.0013 (8)	−0.0036 (9)
O8	0.0186 (9)	0.0184 (10)	0.0213 (11)	0.0065 (8)	0.0053 (8)	0.0022 (8)
O9	0.0330 (12)	0.0234 (11)	0.0267 (13)	0.0084 (9)	−0.0005 (9)	0.0044 (10)
O10	0.0558 (16)	0.0426 (15)	0.0303 (14)	0.0106 (13)	0.0049 (12)	0.0044 (12)
C1	0.0116 (12)	0.0135 (13)	0.0230 (16)	−0.0008 (10)	0.0000 (11)	0.0018 (12)
C2	0.0172 (13)	0.0157 (13)	0.0198 (15)	0.0031 (11)	−0.0012 (11)	−0.0003 (11)
C3	0.0213 (14)	0.0315 (17)	0.0276 (18)	0.0070 (13)	0.0027 (13)	−0.0010 (14)
C4	0.0345 (18)	0.041 (2)	0.0199 (17)	0.0101 (15)	0.0023 (13)	−0.0011 (15)
C5	0.0356 (18)	0.043 (2)	0.0197 (17)	0.0106 (15)	−0.0081 (14)	−0.0037 (15)
C6	0.0212 (14)	0.0410 (19)	0.0272 (18)	0.0119 (13)	−0.0062 (12)	−0.0037 (15)
C7	0.0169 (13)	0.0144 (13)	0.0238 (16)	0.0038 (11)	0.0010 (11)	0.0003 (12)
C8	0.0085 (11)	0.0192 (14)	0.0243 (16)	0.0046 (10)	−0.0050 (10)	−0.0009 (12)
C9	0.0193 (13)	0.0128 (13)	0.0194 (15)	0.0072 (11)	0.0027 (11)	−0.0014 (11)
C10	0.0169 (13)	0.0180 (14)	0.0193 (15)	0.0035 (11)	−0.0010 (11)	−0.0004 (12)
C11	0.0282 (16)	0.0227 (16)	0.0287 (18)	−0.0007 (13)	−0.0039 (13)	−0.0041 (14)
C12	0.0386 (19)	0.038 (2)	0.0212 (18)	0.0048 (15)	−0.0021 (14)	−0.0069 (15)
C13	0.0440 (19)	0.0368 (19)	0.0199 (18)	0.0057 (16)	0.0035 (15)	0.0068 (15)
C14	0.0295 (16)	0.0211 (15)	0.0293 (18)	0.0019 (13)	0.0020 (13)	0.0021 (14)
C15	0.0144 (12)	0.0196 (14)	0.0199 (15)	0.0043 (11)	0.0018 (11)	−0.0003 (12)
C16	0.0172 (13)	0.0129 (13)	0.0194 (15)	0.0071 (10)	−0.0019 (11)	0.0023 (11)
C17	0.0385 (19)	0.037 (2)	0.039 (2)	0.0135 (16)	0.0002 (16)	−0.0016 (17)

### Geometric parameters (Å, °)

**Table:**

Sr1—O3^i^	2.641 (2)	O6—C9	1.251 (3)
Sr1—O3^ii^	2.641 (2)	O6—Sr3^iii^	2.6281 (19)
Sr1—O2^iii^	2.661 (2)	O7—C16	1.248 (3)
Sr1—O2	2.661 (2)	O7—Sr2^iv^	2.6335 (18)
Sr1—O6^iii^	2.6729 (19)	O7—Sr3^viii^	2.729 (2)
Sr1—O6	2.6729 (19)	O8—C16	1.277 (3)
Sr1—O1	2.7742 (17)	O8—Sr2^v^	2.6710 (18)
Sr1—O1^iii^	2.7742 (17)	O8—Sr3^viii^	2.9331 (19)
Sr1—O5^iii^	2.8848 (19)	O9—C17	1.262 (4)
Sr1—O5	2.8848 (19)	O9—Sr3^viii^	2.467 (2)
Sr1—O4^i^	2.9242 (19)	O10—C17	1.256 (4)
Sr1—O4^ii^	2.9242 (19)	C1—C2	1.504 (4)
Sr2—O5	2.536 (2)	C2—C3	1.384 (4)
Sr2—O10	2.556 (2)	C2—C7	1.396 (4)
Sr2—O2	2.6087 (19)	C3—C4	1.384 (4)
Sr2—O9	2.620 (2)	C3—H3A	0.9300
Sr2—O7^iv^	2.6335 (18)	C4—C5	1.378 (4)
Sr2—O8^v^	2.6710 (18)	C4—H4A	0.9300
Sr2—O8	2.6766 (18)	C5—C6	1.384 (5)
Sr2—O4^ii^	2.6789 (18)	C5—H5A	0.9300
Sr3—O9^vi^	2.467 (2)	C6—C7	1.383 (4)
Sr3—O1^i^	2.6128 (19)	C6—H6A	0.9300
Sr3—O6^iii^	2.6281 (19)	C7—C8	1.502 (4)
Sr3—O3	2.6314 (19)	C8—Sr1^vii^	3.125 (3)
Sr3—O4^i^	2.6938 (19)	C8—Sr3^i^	3.420 (3)
Sr3—O5^i^	2.7104 (18)	C9—C10	1.490 (4)
Sr3—O7^vi^	2.729 (2)	C10—C11	1.383 (4)
Sr3—O1	2.8361 (19)	C10—C15	1.400 (4)
Sr3—O8^vi^	2.9332 (19)	C11—C12	1.382 (4)
O1—C1	1.271 (3)	C11—H11A	0.9300
O1—Sr3^i^	2.6129 (19)	C12—C13	1.376 (5)
O2—C1	1.255 (3)	C12—H12A	0.9300
O3—C8	1.250 (3)	C13—C14	1.380 (4)
O3—Sr1^vii^	2.641 (2)	C13—H13A	0.9300
O4—C8	1.264 (3)	C14—C15	1.382 (4)
O4—Sr2^vii^	2.6789 (18)	C14—H14A	0.9300
O4—Sr3^i^	2.6938 (19)	C15—C16	1.510 (4)
O4—Sr1^vii^	2.9242 (19)	C16—Sr3^viii^	3.190 (3)
O5—C9	1.275 (3)	C17—H17A	0.9300
O5—Sr3^i^	2.7104 (18)		
			
O3^i^—Sr1—O3^ii^	180.00 (9)	O3—Sr3—O1	65.33 (5)
O3^i^—Sr1—O2^iii^	72.69 (6)	O4^i^—Sr3—O1	76.04 (6)
O3^ii^—Sr1—O2^iii^	107.31 (6)	O5^i^—Sr3—O1	124.22 (5)
O3^i^—Sr1—O2	107.31 (6)	O7^vi^—Sr3—O1	141.68 (6)
O3^ii^—Sr1—O2	72.69 (6)	O9^vi^—Sr3—O8^vi^	71.45 (6)
O2^iii^—Sr1—O2	180.0	O1^i^—Sr3—O8^vi^	109.25 (5)
O3^i^—Sr1—O6^iii^	103.66 (6)	O6^iii^—Sr3—O8^vi^	82.40 (6)
O3^ii^—Sr1—O6^iii^	76.34 (6)	O3—Sr3—O8^vi^	161.74 (5)
O2^iii^—Sr1—O6^iii^	102.22 (6)	O4^i^—Sr3—O8^vi^	62.49 (5)
O2—Sr1—O6^iii^	77.78 (6)	O5^i^—Sr3—O8^vi^	100.39 (5)
O3^i^—Sr1—O6	76.34 (6)	O7^vi^—Sr3—O8^vi^	45.82 (5)
O3^ii^—Sr1—O6	103.66 (6)	O1—Sr3—O8^vi^	132.73 (5)
O2^iii^—Sr1—O6	77.78 (6)	O9^vi^—Sr3—C16^vi^	86.49 (7)
O2—Sr1—O6	102.22 (6)	O1^i^—Sr3—C16^vi^	89.85 (6)
O6^iii^—Sr1—O6	180.0	O6^iii^—Sr3—C16^vi^	104.15 (6)
O3^i^—Sr1—O1	68.69 (6)	O3—Sr3—C16^vi^	142.52 (6)
O3^ii^—Sr1—O1	111.31 (6)	O4^i^—Sr3—C16^vi^	63.56 (6)
O2^iii^—Sr1—O1	131.93 (5)	O5^i^—Sr3—C16^vi^	83.02 (6)
O2—Sr1—O1	48.07 (5)	O7^vi^—Sr3—C16^vi^	22.65 (6)
O6^iii^—Sr1—O1	61.89 (6)	O1—Sr3—C16^vi^	139.48 (6)
O6—Sr1—O1	118.11 (6)	O8^vi^—Sr3—C16^vi^	23.60 (6)
O3^i^—Sr1—O1^iii^	111.31 (6)	O9^vi^—Sr3—C8^i^	143.07 (7)
O3^ii^—Sr1—O1^iii^	68.69 (6)	O1^i^—Sr3—C8^i^	48.65 (6)
O2^iii^—Sr1—O1^iii^	48.07 (5)	O6^iii^—Sr3—C8^i^	81.07 (6)
O2—Sr1—O1^iii^	131.93 (5)	O3—Sr3—C8^i^	111.74 (6)
O6^iii^—Sr1—O1^iii^	118.11 (6)	O4^i^—Sr3—C8^i^	19.62 (6)
O6—Sr1—O1^iii^	61.89 (6)	O5^i^—Sr3—C8^i^	119.24 (6)
O1—Sr1—O1^iii^	180.0	O7^vi^—Sr3—C8^i^	77.15 (6)
O3^i^—Sr1—O5^iii^	120.83 (6)	O1—Sr3—C8^i^	66.95 (6)
O3^ii^—Sr1—O5^iii^	59.17 (6)	O8^vi^—Sr3—C8^i^	78.75 (6)
O2^iii^—Sr1—O5^iii^	68.95 (6)	C16^vi^—Sr3—C8^i^	73.62 (6)
O2—Sr1—O5^iii^	111.05 (6)	O9^vi^—Sr3—Sr1^vii^	125.58 (5)
O6^iii^—Sr1—O5^iii^	46.67 (5)	O1^i^—Sr3—Sr1^vii^	41.76 (4)
O6—Sr1—O5^iii^	133.33 (5)	O6^iii^—Sr3—Sr1^vii^	144.06 (4)
O1—Sr1—O5^iii^	108.47 (5)	O3—Sr3—Sr1^vii^	38.86 (4)
O1^iii^—Sr1—O5^iii^	71.53 (5)	O4^i^—Sr3—Sr1^vii^	109.12 (4)
O3^i^—Sr1—O5	59.17 (6)	O5^i^—Sr3—Sr1^vii^	44.41 (4)
O3^ii^—Sr1—O5	120.83 (6)	O7^vi^—Sr3—Sr1^vii^	84.43 (4)
O2^iii^—Sr1—O5	111.05 (6)	O1—Sr3—Sr1^vii^	82.91 (4)
O2—Sr1—O5	68.95 (6)	O8^vi^—Sr3—Sr1^vii^	130.22 (4)
O6^iii^—Sr1—O5	133.33 (5)	C16^vi^—Sr3—Sr1^vii^	106.72 (5)
O6—Sr1—O5	46.67 (5)	C8^i^—Sr3—Sr1^vii^	90.35 (4)
O1—Sr1—O5	71.53 (5)	C1—O1—Sr3^i^	119.45 (16)
O1^iii^—Sr1—O5	108.47 (5)	C1—O1—Sr1	89.98 (14)
O5^iii^—Sr1—O5	180.0	Sr3^i^—O1—Sr1	99.39 (6)
O3^i^—Sr1—O4^i^	46.64 (5)	C1—O1—Sr3	129.32 (16)
O3^ii^—Sr1—O4^i^	133.36 (5)	Sr3^i^—O1—Sr3	108.81 (6)
O2^iii^—Sr1—O4^i^	59.32 (5)	Sr1—O1—Sr3	96.94 (5)
O2—Sr1—O4^i^	120.68 (5)	C1—O2—Sr2	126.39 (17)
O6^iii^—Sr1—O4^i^	65.35 (6)	C1—O2—Sr1	95.62 (17)
O6—Sr1—O4^i^	114.65 (6)	Sr2—O2—Sr1	98.51 (6)
O1—Sr1—O4^i^	73.41 (5)	C8—O3—Sr3	121.66 (16)
O1^iii^—Sr1—O4^i^	106.59 (5)	C8—O3—Sr1^vii^	100.71 (17)
O5^iii^—Sr1—O4^i^	75.06 (5)	Sr3—O3—Sr1^vii^	102.43 (7)
O5—Sr1—O4^i^	104.94 (5)	C8—O4—Sr2^vii^	135.86 (16)
O3^i^—Sr1—O4^ii^	133.36 (5)	C8—O4—Sr3^i^	114.66 (15)
O3^ii^—Sr1—O4^ii^	46.64 (5)	Sr2^vii^—O4—Sr3^i^	109.39 (6)
O2^iii^—Sr1—O4^ii^	120.68 (5)	C8—O4—Sr1^vii^	87.01 (16)
O2—Sr1—O4^ii^	59.32 (5)	Sr2^vii^—O4—Sr1^vii^	90.77 (5)
O6^iii^—Sr1—O4^ii^	114.65 (6)	Sr3^i^—O4—Sr1^vii^	96.68 (6)
O6—Sr1—O4^ii^	65.35 (6)	C9—O5—Sr2	112.02 (16)
O1—Sr1—O4^ii^	106.59 (5)	C9—O5—Sr3^i^	132.22 (17)
O1^iii^—Sr1—O4^ii^	73.41 (5)	Sr2—O5—Sr3^i^	115.44 (7)
O5^iii^—Sr1—O4^ii^	104.94 (5)	C9—O5—Sr1	86.87 (15)
O5—Sr1—O4^ii^	75.06 (5)	Sr2—O5—Sr1	94.66 (6)
O4^i^—Sr1—O4^ii^	180.00 (5)	Sr3^i^—O5—Sr1	94.48 (5)
O5—Sr2—O10	153.40 (7)	C9—O6—Sr3^iii^	138.19 (17)
O5—Sr2—O2	75.35 (6)	C9—O6—Sr1	97.19 (16)
O10—Sr2—O2	88.68 (7)	Sr3^iii^—O6—Sr1	104.80 (7)
O5—Sr2—O9	125.53 (6)	C16—O7—Sr2^iv^	143.91 (17)
O10—Sr2—O9	50.80 (7)	C16—O7—Sr3^viii^	99.92 (17)
O2—Sr2—O9	128.29 (6)	Sr2^iv^—O7—Sr3^viii^	111.61 (7)
O5—Sr2—O7^iv^	66.86 (6)	C16—O8—Sr2^v^	118.00 (16)
O10—Sr2—O7^iv^	88.28 (7)	C16—O8—Sr2	121.54 (15)
O2—Sr2—O7^iv^	72.55 (6)	Sr2^v^—O8—Sr2	115.75 (6)
O9—Sr2—O7^iv^	75.32 (6)	C16—O8—Sr3^viii^	89.57 (15)
O5—Sr2—O8^v^	101.16 (6)	Sr2^v^—O8—Sr3^viii^	102.86 (6)
O10—Sr2—O8^v^	105.39 (7)	Sr2—O8—Sr3^viii^	99.56 (6)
O2—Sr2—O8^v^	129.55 (6)	C17—O9—Sr3^viii^	153.6 (2)
O9—Sr2—O8^v^	95.28 (6)	C17—O9—Sr2	91.1 (2)
O7^iv^—Sr2—O8^v^	153.14 (6)	Sr3^viii^—O9—Sr2	114.79 (8)
O5—Sr2—O8	68.06 (6)	C17—O10—Sr2	94.3 (2)
O10—Sr2—O8	123.11 (7)	O2—C1—O1	122.6 (3)
O2—Sr2—O8	143.15 (6)	O2—C1—C2	118.3 (3)
O9—Sr2—O8	73.66 (6)	O1—C1—C2	119.0 (2)
O7^iv^—Sr2—O8	88.90 (6)	O2—C1—Sr1	60.21 (14)
O8^v^—Sr2—O8	64.25 (6)	O1—C1—Sr1	65.40 (14)
O5—Sr2—O4^ii^	85.44 (6)	C2—C1—Sr1	162.29 (18)
O10—Sr2—O4^ii^	106.46 (7)	C3—C2—C7	119.5 (3)
O2—Sr2—O4^ii^	63.25 (6)	C3—C2—C1	119.7 (2)
O9—Sr2—O4^ii^	147.58 (6)	C7—C2—C1	120.6 (3)
O7^iv^—Sr2—O4^ii^	132.53 (6)	C2—C3—C4	120.5 (3)
O8^v^—Sr2—O4^ii^	66.31 (6)	C2—C3—H3A	119.7
O8—Sr2—O4^ii^	116.43 (6)	C4—C3—H3A	119.7
O5—Sr2—C17	144.39 (8)	C5—C4—C3	119.8 (3)
O10—Sr2—C17	25.29 (8)	C5—C4—H4A	120.1
O2—Sr2—C17	108.87 (8)	C3—C4—H4A	120.1
O9—Sr2—C17	25.52 (8)	C4—C5—C6	120.3 (3)
O7^iv^—Sr2—C17	80.49 (8)	C4—C5—H5A	119.9
O8^v^—Sr2—C17	101.96 (8)	C6—C5—H5A	119.9
O8—Sr2—C17	98.66 (8)	C7—C6—C5	120.2 (3)
O4^ii^—Sr2—C17	128.81 (8)	C7—C6—H6A	119.9
O5—Sr2—C9	21.42 (6)	C5—C6—H6A	119.9
O10—Sr2—C9	174.73 (7)	C6—C7—C2	119.7 (3)
O2—Sr2—C9	87.44 (6)	C6—C7—C8	119.3 (2)
O9—Sr2—C9	130.15 (7)	C2—C7—C8	121.0 (3)
O7^iv^—Sr2—C9	87.16 (6)	O3—C8—O4	123.9 (3)
O8^v^—Sr2—C9	79.84 (6)	O3—C8—C7	117.3 (2)
O8—Sr2—C9	59.46 (6)	O4—C8—C7	118.8 (2)
O4^ii^—Sr2—C9	74.88 (6)	O3—C8—Sr1^vii^	56.14 (14)
C17—Sr2—C9	155.22 (9)	O4—C8—Sr1^vii^	69.17 (15)
O5—Sr2—Sr1	46.07 (4)	C7—C8—Sr1^vii^	164.05 (18)
O10—Sr2—Sr1	127.95 (6)	O3—C8—Sr3^i^	108.26 (18)
O2—Sr2—Sr1	41.23 (4)	O4—C8—Sr3^i^	45.72 (12)
O9—Sr2—Sr1	163.24 (5)	C7—C8—Sr3^i^	115.95 (16)
O7^iv^—Sr2—Sr1	88.05 (4)	Sr1^vii^—C8—Sr3^i^	79.71 (6)
O8^v^—Sr2—Sr1	100.66 (4)	O6—C9—O5	122.1 (3)
O8—Sr2—Sr1	108.71 (4)	O6—C9—C10	119.3 (2)
O4^ii^—Sr2—Sr1	47.09 (4)	O5—C9—C10	118.6 (2)
C17—Sr2—Sr1	150.09 (7)	O6—C9—Sr1	59.12 (13)
C9—Sr2—Sr1	49.25 (5)	O5—C9—Sr1	68.79 (14)
O5—Sr2—Sr3^viii^	99.78 (4)	C10—C9—Sr1	153.70 (18)
O10—Sr2—Sr3^viii^	82.14 (6)	O6—C9—Sr2	97.14 (17)
O2—Sr2—Sr3^viii^	147.27 (4)	O5—C9—Sr2	46.56 (13)
O9—Sr2—Sr3^viii^	31.50 (5)	C10—C9—Sr2	126.17 (17)
O7^iv^—Sr2—Sr3^viii^	75.82 (4)	Sr1—C9—Sr2	78.21 (6)
O8^v^—Sr2—Sr3^viii^	83.16 (4)	C11—C10—C15	119.4 (3)
O8—Sr2—Sr3^viii^	42.44 (4)	C11—C10—C9	118.8 (3)
O4^ii^—Sr2—Sr3^viii^	149.44 (4)	C15—C10—C9	121.7 (2)
C17—Sr2—Sr3^viii^	56.95 (7)	C12—C11—C10	121.0 (3)
C9—Sr2—Sr3^viii^	99.28 (5)	C12—C11—H11A	119.5
Sr1—Sr2—Sr3^viii^	145.840 (8)	C10—C11—H11A	119.5
O9^vi^—Sr3—O1^i^	164.51 (6)	C13—C12—C11	119.4 (3)
O9^vi^—Sr3—O6^iii^	73.93 (6)	C13—C12—H12A	120.3
O1^i^—Sr3—O6^iii^	121.54 (6)	C11—C12—H12A	120.3
O9^vi^—Sr3—O3	103.04 (7)	C12—C13—C14	120.3 (3)
O1^i^—Sr3—O3	71.31 (6)	C12—C13—H13A	119.9
O6^iii^—Sr3—O3	113.33 (6)	C14—C13—H13A	119.9
O9^vi^—Sr3—O4^i^	123.48 (6)	C13—C14—C15	120.9 (3)
O1^i^—Sr3—O4^i^	67.42 (6)	C13—C14—H14A	119.6
O6^iii^—Sr3—O4^i^	69.37 (6)	C15—C14—H14A	119.6
O3—Sr3—O4^i^	130.36 (6)	C14—C15—C10	119.0 (3)
O9^vi^—Sr3—O5^i^	87.78 (6)	C14—C15—C16	118.4 (2)
O1^i^—Sr3—O5^i^	76.83 (6)	C10—C15—C16	122.4 (2)
O6^iii^—Sr3—O5^i^	159.68 (6)	O7—C16—O8	122.4 (3)
O3—Sr3—O5^i^	61.60 (6)	O7—C16—C15	120.0 (2)
O4^i^—Sr3—O5^i^	129.85 (6)	O8—C16—C15	117.5 (2)
O9^vi^—Sr3—O7^vi^	96.29 (6)	O7—C16—Sr3^viii^	57.43 (14)
O1^i^—Sr3—O7^vi^	75.32 (6)	O8—C16—Sr3^viii^	66.83 (14)
O6^iii^—Sr3—O7^vi^	126.54 (6)	C15—C16—Sr3^viii^	161.68 (17)
O3—Sr3—O7^vi^	120.04 (6)	O10—C17—O9	123.8 (3)
O4^i^—Sr3—O7^vi^	74.30 (6)	O10—C17—Sr2	60.42 (19)
O5^i^—Sr3—O7^vi^	63.16 (6)	O9—C17—Sr2	63.39 (18)
O9^vi^—Sr3—O1	120.34 (6)	O10—C17—H17A	118.1
O1^i^—Sr3—O1	71.19 (6)	O9—C17—H17A	118.1
O6^iii^—Sr3—O1	61.57 (5)	Sr2—C17—H17A	177.4

Symmetry codes: (i) −*x*+1, −*y*, −*z*; (ii) *x*+1, *y*, *z*; (iii) −*x*+2, −*y*, −*z*; (iv) −*x*+1, −*y*−1, −*z*; (v) −*x*+2, −*y*−1, −*z*; (vi) *x*, *y*+1, *z*; (vii) *x*−1, *y*, *z*; (viii) *x*, *y*−1, *z*.
